# Editorial: Electrical and Structural Remodelling in Atrial Fibrillation: Phenotyping for Personalized Therapy

**DOI:** 10.3389/fphys.2021.697536

**Published:** 2021-05-27

**Authors:** Vijay S. Chauhan, Atul Verma, Sanjiv M. Narayan

**Affiliations:** ^1^Division of Cardiology, Peter Munk Cardiac Center, University Health Network, Toronto, ON, Canada; ^2^Southlake Regional Health Center, Newmarket, ON, Canada; ^3^Stanford University, Palo Alto, Stanford, CA, United States

**Keywords:** atrial fibrillation, structural remodelling, electrical remodelling, fibrotic atrial myopathy, personalized medicine, catheter ablation

Atrial fibrillation (AF) is the most prevalent arrhythmia worldwide, afflicting millions of patients (Chugh et al., 2014). Unfortunately, the success of AF therapy remains modest, in part due to the challenges of translating advances in basic science to the bedside. Atrial remodelling is a widely acknowledged process that accelerates the susceptibility to and progression of AF, and comprises electrical and structural components (Nattel et al., 2008). An increasingly recognized structural component is fibrotic atrial myopathy (FAM), which describes interstitial and replacement fibrosis. However, it is unclear which clinical tools best define remodelling, and whether electrical and structural components progress independently or in concert. Delineation of electrical and structural remodelling of the atria may be central to breakthroughs in therapy and the foundation to tailor therapy for personalized medicine.

This Research Topic focuses on original research and reviews on the clinical implications of electrical and structural remodelling in AF. The 10 contributions cover cutting edge and emerging content areas. This includes electrical remodelling, delineated by clinical AF mapping or translational models. Structurally, this includes characterization of abnormalities including FAM. These contributions provide a foundation for personalized therapy of AF. In this editorial, each contribution is summarized along with its physiologic and clinical implications.

The importance of local atrial electrogram features to delineate FAM and its effect on pulmonary vein isolation (PVI) was investigated by Garg et al. In 20 patients with paroxysmal AF undergoing PVI, left atrial bipolar voltage mapping during sinus rhythm revealed lower global voltage and lower local voltage in those who failed to achieve first pass PVI. Their findings suggest that more global and segmental fibrosis may reduce the success of PVI with radiofrequency energy. Nairn et al. also performed high-resolution left atrial voltage mapping in 28 patients before AF catheter ablation. Bipolar and unipolar voltages were compared and were found to highly correlate both in sinus rhythm and AF. A unipolar voltage threshold was computed based on a linear transformation of bipolar voltage that provided high spatial concordance. The authors report that reduced bipolar inter-electrode distance from 6 to 2 mm did not significantly increase the correlation between mapped areas of low voltage compared to unipolar recordings, and suggested that spatial resolution may not be a clinically significant confounder of mapping low voltage aspects of FAM when using contemporary tools. In eight patients with persistent AF, Ali et al. evaluated left atrial FAM using late gadolinium enhanced magnetic resonance imaging (LGE MRI) and local conduction velocities as defined by bipolar activation mapping in sinus rhythm. They found that LGE MRI signal intensity correlated with colocalized conduction velocity, but only at spatial scaling of a mapping catheter electrode. This finding extends work by Caixal et al. (2021) who recently showed that LGE MRI signal intensity correlated with atrial conduction velocity in sinus rhythm unless the atrium was markedly dilated. These studies suggest that LGE MRI may provide functional assessment of FAM in terms of conduction slowing and predisposition to reentry. Roney et al. constructed 50 AF patient-specific left atrial bi-layer models incorporating FAM based on their LGE MRIs and simulated AF in order to determine the location of electrical drivers. Several different ablation approaches were tested, including PVI, linear ablations, FAM ablation, driver ablation, or a combination thereof to determine the optimal approach to terminate AF or convert AF to an atrial tachycardia. The study demonstrated the need for a patient-specific ablation strategy based on their FAM and driver distribution.

The mechanisms of conditional AF structural modelling were further explored in two mechanistic studies. In a mouse model of exercise-induced AF, Oh et al. used transcriptomic bioinformatic analysis of atria to investigate novel collagen pathways that increase AF vulnerability. During 6 weeks of exercise, there was differential regulation of atrial genes linked to mechanosensing, extracellular matrix and tumor necrosis factor pathways, which was 2-fold higher than in the ventricles. Transcriptomics were temporally dynamic and related to increased preload and atrial stretch seen with exercise. Tang et al. also studied molecular modulation of structural remodelling, in particular the regulation of angiotensin 2-induced atrial fibrosis. In rats treated for 2-weeks with apelin, an inhibitor of fibrosis, there was a decrease in angiotensin 2-induced atrial fibrosis and AF inducibility with programmed stimulation. The protective effects of apelin were mediated by suppression of Smad2-dependent fibrosis, which may provide an effective up-stream therapy for atrial fibrosis and AF.

With respect to characterizing electrical modelling, Rodrigo et al. evaluated regional differences in activation rates during AF using non-invasive electrocardiographic imaging (ECGI) and contact mapping. ECGI provided a moderate estimation of intracardiac recording-based activation rates in AF after filtering by high spectral organization. The ECGI-derived highest dominant frequency predicted acute AF ablation success suggesting that this approach may provide an effective tool to identify patient responders for personalized ablation therapy. The importance of discerning repetitive AF propagation as putative targets for catheter ablation was also studied by Zeemering et al. These AF patterns were automatically identified from high-density-electrode array recordings in goat model of AF using a novel computational tool based on recurrence plots. Activation breakthrough and reentry were seen as repetitive conduction patterns which became shorter with more prolonged AF duration. Using this methodology with high-resolution clinical mapping catheters may delineate putative targets for AF catheter ablation.

The final two contributions by Ho et al. and Ng et al. provide a comprehensive review of electrophenotyping to improve our understanding of patient-specific AF mechanisms than may guide personalized therapy. This is necessary because AF mechanisms and propagation is dependent on the extent of structural and electrical remodelling, which can vary even among patients with comparable AF risk factors, AF duration, and cardiovascular disease.

In summary, improved therapy for AF is likely to require better delineation of FAM in individual patients, both in its structural and electrical components. Structural FAM is now widely appreciated, yet it is undefined whether to identify this by non-invasive imaging, by voltage mapping or via its functional effects such as conduction slowing. It is equally undefined how to treat structural FAM. Electrically, it is critical to delineate how FAM leads to functional arrhythmias. Identification of patient-specific AF triggers or AF sustaining substrates, such as localized drivers, is limited by the spatial resolution of clinical tools and lack of accepted gold standards for validation. Electrophenotyping of patients with AF may enable us to identify those in whom therapy should target FAM, and those in whom FAM and AF may progress and who may instead benefit from more general strategies. The 10 articles in this Research Topic add to our understanding of FAM, and provide exciting areas for further investigation. We congratulate the authors.

## Author Contributions

All authors contributed to content and critical review of this editorial.

## Conflict of Interest

The authors declare that the research was conducted in the absence of any commercial or financial relationships that could be construed as a potential conflict of interest.

## References

[B1] Caixal G. Alarcon F. Althoff T. Nunez-Garcia M. Benito E. Borras R.. (2021). Accuracy of left atrial fibrosis detection with cardiac magnetic resonance: correlation of late gadolinium enhancement with endocardial voltage and conduction velocity. Europace 23, 380–388. 10.1093/europace/euaa313 33227129

[B2] Chugh S. Havmoeller R. Narayanan K. Singh D. Rienstra M. Benjamin E.. (2014). Worldwide epidemiology of atrial fibrillation: a global burden of disease 2010 study. Circulation 129, 837–847. 10.1161/CIRCULATIONAHA.113.005119 24345399 PMC4151302

[B3] Nattel S. Burstein B. Dobrev D. (2008). Atrial Remodeling and Atrial Fibrillation: Mechanisms and Implications. Circ. Arrhythmia Electrophysiol. 1, 62–73. 10.1161/CIRCEP.107.754564 19808395

